# 70740 Impact of fundus pigmentation on retinal layer visibility on investigational bedside optical coherence tomography in preterm infants

**DOI:** 10.1017/cts.2021.496

**Published:** 2021-03-30

**Authors:** Kai R. Seely, Michelle McCall, Brendan McGeehan, Katrina Winter, Du Tran-Viet, Vincent Tai, Gui-Shuang Ying, Cynthia A. Toth

**Affiliations:** 1 Duke University; 2 University of Pennsylvania

## Abstract

ABSTRACT IMPACT: This study helps translate investigational bedside optical coherence tomography into improved diagnosis and care of preterm infants at risk for retinopathy of prematurity. OBJECTIVES/GOALS: In retinopathy of prematurity screening, fundus photography may be of limited quality in eyes with dark fundus pigmentation (FP). The goal of this study was to evaluate the impact of FP on overall scan quality and retinal layer visibility on investigational bedside optical coherence tomography (OCT) in preterm infants. METHODS/STUDY POPULATION: We analyzed 846 OCT scans captured prospectively from 188 eyes of 94 preterm infants enrolled in the BabySTEPS study (NCT02887157). Trained ophthalmologists, masked to OCT findings, determined FP (blond, medium, or dark). Expert graders, masked to FP, evaluated OCT images for: 1) overall OCT quality (excellent, acceptable, poor, or unusable); and 2) all age-appropriate retinal layers visible (yes or no). To assess the association of FP with OCT quality (excellent/acceptable or poor/unusable) and retinal layer visibility, we performed multivariable logistic regression modeling, adjusting for biologic and demographic confounders and correlations from repeated OCT scans and paired eyes. RESULTS/ANTICIPATED RESULTS: Mean birthweight was 964 (SD=283) grams, mean gestational age was 27.8 (SD=2.6) weeks, 48 (51%) infants were male, and 51 (54%) were non-white. On exam, 72 (38%) eyes had blond FP, 92 (49%) had medium, and 24 (13%) had dark. OCT quality was excellent or acceptable in 725 scans (86%) and all age-appropriate retinal layers were visible in 781 scans (92%). Compared to eyes with blond FP, eyes with medium and dark FP did not have higher odds of poor/unusable OCT scan quality (adjusted OR 0.87 [95% CI 0.50-1.48] and 0.49 [95% CI 0.16-1.55], respectively) or not all age-appropriate retinal layers visible on OCT (adjusted OR 1.17 [95% CI 0.39-3.51] and 0.57 [95% CI 0.15-2.20], respectively). DISCUSSION/SIGNIFICANCE OF FINDINGS: Medium and dark FP did not impact overall scan quality or age-appropriate retinal layer visibility on investigational bedside OCT in preterm infants. This study supports the feasibility of using OCT to analyze retinal microanatomy in diverse populations of preterm infants with a range of FP.

